# First records of two mealybug species in Brazil and new potential pests of papaya and coffee

**DOI:** 10.1673/2006_06_23.1

**Published:** 2006-09-28

**Authors:** Mark P. Culik, David dos Santos Martins, Penny J. Gullan

**Affiliations:** 1 Instituto Capixaba de Pesquisa, Assistência Técnica e Extensão Rural – INCAPER, Rua Afonso Sarlo 160, CEP 29052-010, Vitória, Espírito Santo, Brasil; 2 Department of Entomology, University of California, One Shields Avenue, Davis, CA 95616-8584, USA

**Keywords:** Dysmicoccus grassii, Ferrisia malvastra, Ferrisia virgata, Phenacoccus tucumanus, Planococcus minor, Plotococcus capixaba, Pseudococcus elisae, Coffea canephora, Carica papaya, Bidens pilosa, Eugenia cf. pitanga

## Abstract

Five mealybug (Hemiptera: Pseudococcidae) plant pest species: Dysmicoccus grassii (Leonardi), Ferrisia malvastra (McDaniel), Ferrisia virgata (Cockerell), Phenacoccus tucumanus Granara de Willink, and Pseudococcus elisae Borchsenius are recorded for the first time in the state of Espírito Santo, Brazil. These are the first records of D. grassii in Brazil, from papaya (Carica papaya, Caricaceae), and from coffee (Coffea canephora, Rubiaceae). Ferrisia malvastra is also newly recorded in Brazil, where it was found on Bidens pilosa (Asteraceae). Ferrisia virgata was collected from an unidentified weed and Phenacoccus tucumanus from Citrus sp. (Rutaceae). Plotococcus capixaba Kondo was found on pitanga (Eugenia cf. pitanga, Myrtaceae) and Pseudococcus elisae on Coffea canephora, which are new host records for these mealybugs.

## Resumo

São registradas pela primeira vez a ocorrência das cochonilhas (Hemiptera: Pseudococcidae) Dysmicoccus grassii (Leonardi), Ferrisia malvastra (McDaniel), Ferrisia virgata (Cockerell), Phenacoccus tucumanus Granara de Willink e Pseudococcus elisae Borchsenius no estado do Espírito Santo. Destacam-se os registros de Dysmicoccus grassii e Ferrisia malvastra que são os primeiros no Brasil, sendo que D. grassii é o primeiro no mamoeiro (Carica papaya, Caricaceae) e em café conilon (Coffea canephora, Rubiaceae). A espécie Ferrisia malvastra foi encontrada em picão preto (Bidens pilosa, Asteraceae), F. virgata coletada de uma planta daninha não identificada, e Phenacoccus tucumanus em limoeiro galego (Citrus sp., Rutaceae). Plotococcus capixaba Kondo foi coletada em pitangueira (Eugenia cf. pitanga, Myrtaceae) e Pseudococcus elisae em Coffea canephora, sendo estas plantas consideradas, respectivamente, como novas hospedeiras dessas espécies de cochonilhas.

## Introduction

Mealybugs (Hemiptera: Pseudococidae) are small, soft-bodied insects that feed by sucking plant sap. Adult females and nymphs are wingless and frequently covered in a white, powdery or mealy wax secretion. In addition, the margin of the body often has a series of white, lateral wax filaments that typically are most prominent posteriorly. Adult males, if present, are short-lived, non-feeding and rarely collected. Some mealybug species cause considerable economic damage to agricultural and horticultural plants (McKenzie 1967; Williams and Granara de Willink 1992; Miller et al. 2002, 2005b). Plant damage by mealybugs results from the direct effects of sap removal and injection of toxins, as well as indirectly by honeydew contamination and associated sooty mold growth that decreases photosynthesis (Mibey 1997), and occasionally from the effects of transmitted plant viruses. Feeding damage may cause leaf yellowing, defoliation, reduced plant growth, and death of plants. The occurrence of honeydew and sooty mold may reduce the marketability of plant products such as fruits. Even if plant damage is not apparent, the mere presence of mealybugs can be a quarantine concern, adding to costs of production to prevent or eliminate their presence on plants and produce.

Papaya (Carica papaya L., Caricaceae) is grown throughout Brazil and cultivation of this fruit is especially important economically in the state of Espírito Santo (ES) (Alves 2003). However, information on the occurrence and distribution of mealybug species on crops such as papaya in Brazil is limited. Worldwide, at least nine species of mealybugs have been recorded as pests of papaya, of which five occur in Brazil (Culik et al. 2003) but none has been recorded previously on papaya in this country (Silva et al. 1968; Medina et al.1989). Furthermore, of 64 mealybug species recorded from Brazil (Ben-Dov et al. 2005), few have previously been identified from Espírito Santo (Culik and Gullan 2005). Therefore, as part of efforts for development of integrated crop production, including papaya, in Brazil (Martins et al. 2003), mealybugs were collected during 2004 and 2005 from various plants in Espírito Santo to identify the species present in this area. Here we document new mealybug records from papaya and other plants in Espírito Santo.

## Materials and methods

Mealybug specimens were collected during surveys of the insect fauna of papaya orchards in Espírito Santo carried out by the Espírito Santo rural research and extension institute INCAPER (Instituto Capixaba de Pesquisa, Assistência Técnica e Extensão Rural) and when noticed on plants during fieldwork or other activities.

All mealybug specimens were slide-mounted for identification using the method outlined in Williams and Granara de Willink (1992), except that xylene was used instead of clove oil. Voucher specimens of these insects are deposited in the arthropod collections of INCAPER, Vitória, Espírito Santo; and the Bohart Museum of Entomology, Department of Entomology, University of California, Davis, California, U.S.A.

## Results

Five mealybug species not reported previously from Espírito Santo were found, as well as a new host for an additional species (Table 1). Dysmicoccus grassii (Leonardi) was first observed infesting C. papaya fruits of seven plants in a commercial orchard in December 2004 and five additional papaya fruits infested with D. grassii were collected from the same location a few days later (eggs, nymphs and males as well as adult female mealybugs were present on the fruit). In addition, D. grassii was obtained from the inflorescence of coffee plants of Coffea canephora (Rubiaceae) in February 2005.

**Table 1:**
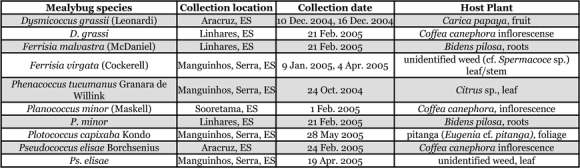
Records of mealybugs (Hemiptera: Pseudococcidae) collected from Espírito Santo (ES), Brazil

A single specimen of Pseudococcus elisae Borchsenius was identified from the inflorescence of C. canephora in February 2005, and an additional specimen of this species was collected from an unidentified weed in another municipality in April 2005. In February 2005, Planococcus minor (Maskell) was collected from the roots of Bidens pilosa growing in a coffee field and was also collected from the inflorescence of C. canephora, confirming the presence of this mealybug species as a potential pest of coffee in Espírito Santo (Santa Cecília et al. 2002).

Specimens of Ferrisia malvastra (McDaniel) were collected from the roots of Bidens pilosa (Asteraceae) growing near a coffee field and two collections of Ferrisia virgata (Cockerell) were made three months apart from the stem or leaves of an unidentified weed from the yard of a home. In the same yard, two specimens of Phenacoccus tucumanus Granara de Willink were taken from foliage of a “limão galego” tree (Citrus sp., Rutaceae) and Plotococcus capixaba Kondo was collected from the foliage of a heavily infested pitanga bush (Eugenia cf. pitanga, Myrtaceae).

Although F. virgata and Ph. tucumanus have been found previously in Brazil (Silva et al. 1968; Williams and Granara de Willink 1992), these are the first records of these species in Espírito Santo, and the first record of Pl. capixaba from pitanga. The records of D. grassii and F. malvastra are the first from Brazil. This is also the first report of D. grassii from papaya, and the first reports of D. grassii and Ps. elisae from C. canephora.

## Discussion

 Dysmicoccus grassii (synonym D. azalon Williams) is considered to be of Neotropical origin, but it was originally described from the Canary Islands (Williams and Granara de Willink 1992). It has been found in Africa, Europe and North America (Ben-Dov 2005a; Miller and Miller 2002; Miller et al. 2005b) and recently was reported for the first time in southern Asia, including on Theobroma cacao (Williams 2004). Although D. grassii is widely distributed in the Neotropics, it has not previously been recorded in Brazil. D. grassii is polyphagous with agriculturally important hosts including mango, pineapple, coffee, and cacao (Ben-Dov 2005a) and it is considered to be a pest of bananas in the Canary Islands (Williams and Granara de Willink 1992) and in Nigeria (Matile-Ferrero and Williams 1995). Watson and Kubiriba (2005) have suggested that it could become more widespread in Africa.

**Figure 1:**
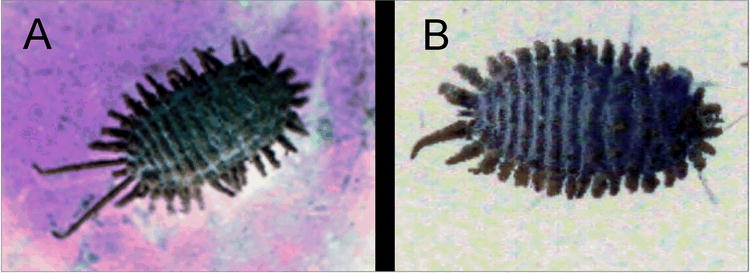
Typical appearance of adult female mealybugs: A, Dysmicoccus grassii on papaya, Aracruz, ES, December 2004; B, Phenacoccus tucumanus from citrus, Serra, ES, 24 October 2004 (photograph, MPC).

The present work is the first record of papaya and C. canephora as hosts for D. grassii. On C. canephora, this mealybug was found in the inflorescence. D. grassii is similar taxonomically to the species D. texensis Tinsley (also recorded as D. bispinosus Beardsley) which occurs from the Neotropics through to the southern USA on a range of hosts (Ben-Dov 2005a), including on the roots of coffee in Brazil (Williams and Granara de Willink 1992; Santa-Cecília et al. 2002). As far as we know, D. grassii has never been collected on the roots of its host plants. On C. papaya, D. grassii was only observed on fruits and other parts of the plants did not appear to be infested. The infestation of D. grassii appeared to be concentrated near the peduncle of the fruits, and yellowing and rot in the area of the peduncle of the unripe (green), mealybug infested papaya fruit also was noted. Eggs, nymphs and males as well as adult female mealybugs were observed on the infested fruit indicating the suitability of papaya as a host for this mealybug. Adult females of D. grassii, as observed in this study (Fig. 1A), are pinkish, densely covered with white, powdery wax and have 16 pairs of short, thick wax filaments (less than a quarter of the length of the body) laterally on the body and one pair of long terminal wax filaments (about half as long as the body).

 Ferrisia virgata (striped mealybug) is found throughout the world on a very broad range of host plants including species in many agriculturally important families such as Cucurbitaceae, Fabaceae (=Leguminosae), Musaceae, Myrtaceae, Arecaceae (=Palmae), Rubiaceae, Rutaceae, and Solanaceae (Ben-Dov 2005a). F. virgata is a common pest on many plants, is a vector of Cacao swollen-shoot badnovirus (CSSV) in Africa and Piper yellow mottle virus (PYMV) in India (Bhat et al. 2003;Ben-Dov 2005a), and is reported to be a pest of C. papaya in Micronesia (Nafus et al. 1999). This species is widespread in Brazil and has been noted previously in the states of Bahia, Pará, Paraíba, Rio Grande do Norte, Rio de Janeiro, and São Paulo (Silva et al. 1968) but apparently it is not known to be an important pest in this country (Anonymous 2003). Many other potential host plants of F. virgata (mango, papaya, pineapple etc.) were present at the site in Manguinhos where F. virgata was collected in this study but, although observations were made on some of these potential hosts and several other mealybug species were found on such plants (Culik and Gullan 2005), F. virgata was observed only on a herbaceous weed in Manguinhos. However, the species was also found on the foliage of a mixed group of potted ornamentals in Vitória, ES, in April 2005. The appearance of live F. virgata (Miller et al. 2005a; Osborne 2005) is distinctive: adult females generally appear grayish, covered with white powdery wax and a pair of dark stripes (or rows of spots) dorsally (as the common name implies), with the body relatively elongate, tapering posteriorly, and terminating in a pair of long wax filaments.

 Ferrisia malvastra (malvastrum mealybug) is a widespread and polyphagous species (Ben-Dov 2005a). Although it is not commonly known to be a significant plant pest, it has been confused with F. virgata and prior to recognition of F. malvastra as distinct from F. virgata, F. malvastra may have been misidentified as F. virgata (Ben-Dov 2005a). It is considered to be a pest in the US (Miller et al. 2002) and is of concern as a potential agricultural pest in Israel (Ben-Dov 2005b). This species is parthenogenetic and formerly was known as the uniparental form of F. virgata and then as F. consobrina Williams and Watson (e.g., Williams and Watson 1988; Williams and Granara de Willink 1992). Live Ferrisia malvastra and F. virgata look very similar, but the former has a more rounded body (PJG, personal observation) and tends to be common on herbs and shrubs (Ben-Dov 2005b). The painting by Mary Foley Benson purported to be an infestation of F. virgata on lantana (McKenzie 1967, color plate X) is probably F. malvastra as the collection data match specimens now known to be F. malvastra and the accompanying taxonomic drawing resembles F. malvastra (PJG, unpublished).

 Phenacoccus tucumanus is known only from South America where it has been found on only a few hosts: several Citrus species, an unidentified weed, and Brazilian pepper, Schinus terebinthifolius (Anacardiaceae) (Williams and Granara de Willink 1992), a plant native to Brazil and cultivated to a limited extent in this country. Adult females of Ph. tucumanus, as observed in this study (Fig. 1B), are densely covered with white, powdery wax, with an oval body surrounded by ~18 pairs of short, thick wax filaments (less than a quarter of the length of the body). Although only a few specimens of Ph. tucumanus were found, this study confirms that this potential pest is present in Espírito Santo.

 Plotococcus capixaba is a species recently described (Kondo et al. 2005) and known only from Espírito Santo and São Paulo, Brazil, on Leandra erinacea (Melastomataceae) and jaboticaba, Myrciaria jaboticaba (Myrtaceae). This record of Pl. capixaba on pitanga confirms that this mealybug is likely to be more widespread and common than has previously been realized. On pitanga, Pl. capixaba has an appearance similar to that observed on jaboticaba. Nymphs and adult females are light yellow in color or covered with white powdery wax, with two long terminal wax filaments, and the insects form extensive clumps of wax on the undersurface of leaves. Eggs are laid under the wax, which apparently serves as protection for eggs and nymphs. Despite the noticeable infestation on pitanga, sooty mold and leaf drop, which appeared to be associated with this insect on jaboticaba (Kondo et al. 2005), was not apparent on pitanga.

 Pseudococcus elisae (banana mealybug) has been recorded previously only from Central and South America, and Florida, and is known from relatively few host plant species (Ben-Dov 2005a; Miller et al. 2005b). However, its hosts do include agriculturally important species such as banana (Musa paradisiaca) and coffee (Coffea arabica) (Williams and Granara de Willink 1992; Gimpel and Miller 1996; Ben-Dov 2005a), and it is considered to be a pest of black pepper in Brazil as a vector of Piper yellow mottle virus (PYMV) (Duarte and Albuquerque 2003), a disease currently not present in Espírito Santo. Ps. elisae has been reported from Coffea sp. in Trinidad and from C. arabica in Colombia (Williams and Granara de Willink 1992) but this is the first record of this species on coffee in Brazil and the first report from C. canephora. Adult females of this mealybug are purplish-gray with the body surrounded by long, thin lateral and terminal wax filaments (Miller et al. 2005a).

Although D. grassii, F. malvastra, F. virgata, Ph. tucumanus, Pl. capixaba, and Ps. elisae are not currently known as important pests in Espírito Santo, the records reported here confirm that these potential pests are present in this state. Knowledge of the presence of these mealybugs may enable producers and researchers to consider the presence of such potential pests in production of crops and development of management methods to avoid practices that may favor development of damaging populations of these pests in the future. Cacao and black pepper are economically important crops in Espírito Santo and these records provide confirmation that F. virgata and Ps. elisae, which are vectors of Cacao swollen-shoot badnovirus and Piper yellow mottle virus, are present in this state, thus reaffirming the importance of quarantine measures to prevent the entry of these diseases to this area. Since D. grassii and Ps. elisae have not been noted previously as pests of papaya and C. canephora, further research on these species and their effects on these economically important crops is especially warranted.
